# Clinical Lecture

**Published:** 1880-09-20

**Authors:** H. Augustus Wilson

**Affiliations:** Philadelphia


### CLINICAL LECTURE.

BY H. AUGUSTUS WILSON, M.D., PHILADELPHIA.

[Reported by John McLean.]

*Cholesterin in Vitreous Humor and Cataract.*—The first case which I bring before you to-day, is Mr. A. H——, aged 74 years. This gentleman comes to me with the following history. Three years ago he fell from a car and was dragged some distance, receiving a severe contusion on the head. One year afterwards his right eye began to fail, and about eight or twelve months later the left also became affected. An examination, with the ophthalmoscope, reveals first of all a number of brilliant specks floating about in the vitreous humor.

This brilliant appearance is due, according to Soelberg Wells, to the presence of crystals of cholesterin in the vitreous humor. Our patient tells us that after sitting quietly with his eyes closed for a half hour or so, if he opens them gradually and looks around, he can distinctly perceive the outlines of the buildings upon the opposite side of the street. The slightest movement, however, serves to set these little bodies in motion, and they, coming before the field of vision, again obscure the sight.

Besides this condition he has in the left eye atrophy of the optic nerve, and enlargement and distortion of the veins, which may be discovered with the ophthalmoscope, when the particles of cholesterin settle down, thus leaving the vitreous humor comparatively clear.

The right eye when examined by oblique illumination, reveals an opacity which I find upon further examination with the ophthalmoscope to be situated in the lens.

Having thus diagnosed a cataract it is important that we should ascertain its character.

There are, as you all know, two kinds of cataract, hard and soft. Soft cataract occurs usually before the thirty-fifth year, while hard, or senile cataract occurs after that period. Again, in soft cataract there are no striæ, while these are always present in the hard variety.

The ophthalmoscope reveals in this case striæ distinctly marked, and this together with the fact that the patient is 74 years old, renders the diagnosis of hard cataract very easy. It was this cataract which induced the patient to seek my advice relative to its removal.

He is very anxious to have an operation performed for the restoration of his sight, and it is for us to study the condition of the eye behind the cataract, that we may form some idea of the advisability of an operation.

If I was able to judge from the other eye that there were no lesions of the optic nerve, retina, or vitreous humor, I might fairly conclude that the cataract was the only cause of his not seeing, but, as I have already told you, the examination of the left eye has revealed optic atrophy, and this very rare condition known as cholestrin in the vitreous humor; and I have every reason for believing that the right eye is similarly affected.

Hence I am compelled to tell our patient that I can offer him no hope whatever from an operation. The treatment in this case has been on the expectant plan.

The patient has been taking potass. iodide gr. x, three times daily for some time, and I am happy to say that the particles of cholesterin have materially decreased in number, but I can see no change whatever in the optic atrophy. I am also using a one-grain solution of sulphate of atropia; partly to give him a wider field of vision, but more especially to make him believe that something is being done for him.

*Purulent Inflammation of the Middle Ear.*—The next case is a little girl, Rebecca G——, aged 12, who has been under treatment for some time. For several years she had a profuse, purulent discharge from the right ear, and was hard of hearing.

The first time I saw her I found upon examination that she had strawberry granulations on the posterior wall of the meatus, just external to the membrana tympani; together with a perforation of the membrana tympani itself.

When she first came to me, her hearing distance was three-eighteenths, that is to say, she heard a watch held three inches from her ear, which a normal ear should hear at eighteen inches. She was treated for some time with monochloracetic acid, but lately I have made weekly applications of nitate of silver, in stick, followed by iodoformized cotton.

The method of preparing the latter for application is as follows:

Make a solution of

| | |
|---|---|
| Iodoform | ʒss |
| Ether | ℥j |
| Oleum gaultheriæ | gtts x |

A piece of absorbent cotton is completely saturated with this solution. The ether is then evaporated, thus leaving the iodoform in a finely divided state, evenly distributed throughout the cotton.

When I wish to make an application, I simply cut off a piece of this prepared cotton, insert it in the meatus and direct the patient to leave it there for twenty-four hours, and then remove it and syringe the ear thoroughly with warm water.

I find this mode of administering iodoform externally far superior to any other with which I am acquainted. I have used it in chronic ulcers of the leg, and have found upon removing the cotton twenty-four hours after application, that the iodoform has been almost entirely absorbed.

My experience in treating purulent inflammation of the ear, has still further shown this to be a valuable mode of administering iodoform externally. Under the above treatment this little girl has improved very much. The discharge has entirely ceased, the granulations have very materially decreased in size, the perforated membrana tympani has healed, and her hearing is now fifteen-eighteenths. I think that after one more week of treatment she will be discharged from the dyspensary.

*Opacity of Cornea with Anterior Synechia.*—The next case is Annie B——, aged six months. Her mother says that for four months she has had chronic purulent ophthalmia, probably originating in opthalmia neonatorum. She has in the right eye prolapse of the iris with anterior synechia; and large, thick, heavy opacity all over the pupillary space. In the left eye the opacity is epithelial in character, very slight, and on the outer surface of cornea.

I bring this case before you to show you what may occur in your own practice, should you allow a case of ophthalmia neonatorum to go untreated.

The child now has great photophobia, or dread of light, which keeps her in the house, and in a dark corner away from the sunshine. In the right eye there has been an ulcer, which, perforating the cornea, allowed the aqueous humor to escape. The iris has been pressed forward, and has been caught in this opening. It is now permanently fast there, and constitutes the condition known as anterior synechia.

The only treatment which will benefit the right eye is iridectomy, which I shall perform when she gets a little older. In the left eye I hope to remove the opacity by very delicate scarification of the epithelial surface, followed by the application of calomel in a fine state of subdivision, which I shall dust into the eye with a camel's hair brush. I must warn you not to use calomel insufflation at the same time that you are giving iodide of potassium internally, for experience has shown that iodide of potassium being readily absorbed, and distributed through the whole system, will come in contact with the calomel in the eye, decomposing it and forming the iodide of mercury, which acting as a caustic sets up severe inflammation of the coujunctiva. The sight in this case, however, can never be entirely restored, as objects will always appear more or less distorted, owing to the broken up condition of the corneal surface.

I am using atropia for the purpose of dilating the pupil beyond the opacity, so that the retina may have the stimulus of light to prevent the occurrence of amblyopia (partial anæsthesia of retina), which is apt to follow disuse.

*Traumatic Injury of Cornea with Prolapse of Iris.*—The last case which I shall have the pleasure of bringing before you to-day is that of a young man, Julius E——, aged eighteen, who about ten days ago was struck in the eye by a piece of steel.

When I first saw him two days ago, the eye was very much inflamed, and the pupillary space was egg-shaped, owing to a prolapse of the iris.

It is impossible at this late stage for me to say whether the piece of steel has perforated the cornea, or whether it has caused an ulcer, which has gone on to perforation, resulting in this prolapse of the iris. The fact that I am unable to find any wound of the iris, or the presence of the piece of steel in the eye, leads me to believe that the latter is the case.

Could I have seen the case early before perforation had taken place, I shouid have tapped the anterior chamber, and thus have relieved the pressure, which was one of the factors in the petforation. The treatment so far has been the application of an eight-grain solution of sulphate of atropia, with a view to drawing the prolapsed iris out of the perforation and relieving the pain.

To-day I shall apply a two-grain solution of sulphate of eserine, and direct him to drop two or three drops of it in the eye several times a day. This will produce tension in a direction opposed to that exerted by the atropia, and I hope in this way to draw the iris completely out of the opening.—*Med. Bulletin.*

